# Supplementary material for: Empirical methods for controlling false positives and estimating confidence in ChIP-Seq peaks
Source: BMC Bioinformatics. 2008 Dec 5;9:523. doi: 10.1186/1471-2105-9-523 (PMC2628906; doi:10.1186/1471-2105-9-523)
Supplement: Additional File 1 — A variety of html documents from the USeq web site detailing the available applications, their best usage, output file type descriptions, command line menus, etc. [file 1471-2105-9-523-S1.zip › USeqUserGuides/JavaDoc.zip!/JavaDoc/trans/main/class-use/BindingPeak.html]

Uses of Class trans.main.BindingPeak


|  |  |  |
| --- | --- | --- |
| **Overview** \| **Package** \| **Class** \| **Use** \| **Tree** \| **Deprecated** \| **Index** \| **Help** | |  |
| PREV   NEXT | **FRAMES**    **NO FRAMES**     **All Classes** |  |


---


## **Uses of Class trans.main.BindingPeak**

| Packages that use BindingPeak | |
| --- | --- |
| **trans.main** | Principle classes and scripts used in TiMAT2 analysis. |

Uses of BindingPeak in trans.main

| Methods in trans.main that return BindingPeak | |
| --- | --- |
| `BindingPeak[]` | `Interval.getBindingPeaks()` |

| Methods in trans.main with parameters of type BindingPeak | |
| --- | --- |
| `static void` | `IntervalGFFPrinter.gff(BindingPeak peak, Interval i, java.lang.String name, int peakNumber, java.lang.StringBuffer s)`             Appends a GFF3 line for a BindingPeak |
| `void` | `Interval.setBindingPeaks(BindingPeak[] bindingPeaks)` |

---


|  |  |  |
| --- | --- | --- |
| **Overview** \| **Package** \| **Class** \| **Use** \| **Tree** \| **Deprecated** \| **Index** \| **Help** | |  |
| PREV   NEXT | **FRAMES**    **NO FRAMES**     **All Classes** |  |


---
